# Future Patient—Telerehabilitation of Patients With Atrial Fibrillation: Protocol for a Multicenter, Mixed Methods, Randomized Controlled Trial

**DOI:** 10.2196/64259

**Published:** 2025-02-18

**Authors:** Birthe Dinesen, Andi Eie Albertsen, Elisabet Dortea Ragnvaldsdóttir Joensen, Helle Spindler, Katja Møller Jensen, Kristian Kidholm, Lars Frost, Lars Dittman, Mathushan Gunasegaram, Søren Paaske Johnsen, Mads Rovsing Jochumsen, Dorthe Svenstrup

**Affiliations:** 1 Laboratory of Welfare Technology - Digital Health and Rehabilitation Department of Health Science and Technology Aalborg Universitet Gistrup Denmark; 2 Department of Cardiology Regional Hospital Viborg Viborg Denmark; 3 Department of Psychology and Behavioral Sciences University of Aarhus Aarhus Denmark; 4 Center for Innovative Medical Technology Odense University Hospital Odense Denmark; 5 Diagnostic Centre University Clinic for Development of Innovative Patient Pathways Silkeborg Regional Hospital Silkeborg Denmark; 6 Department of Electrical and Photonics Engineering Technical University of Denmark Copenhagen Denmark; 7 Danish Center for Health Services Research Department of Clinical Medicine Aalborg University Hospital & Aalborg University Aalborg Denmark; 8 Neural Engineering and Neurophysiology, Center for Rehabilitation Robotics Department of Health Science and Technology Aalborg University Aalborg Denmark

**Keywords:** atrial fibrillation, telerehabilitation, quality of life, research design, patient education, co-creation, randomized controlled trial, chronic, cardiovascular disease, adult, aging, prevalence, comorbidity, Future Patient, patient engagement, primary outcome, cost-effectiveness, monitoring, health care professional, digital health, remote therapy, telehealth

## Abstract

**Background:**

Atrial fibrillation (AF) is a chronic cardiovascular condition with a lifetime risk of 1 in 3 and a prevalence of 3% among adults. AF’s prevalence is predicted to more than double during the next 20 years due to better detection, increasing comorbidities, and an aging population. Due to increased AF prevalence, telerehabilitation has been developed to enhance patient engagement, health care accessibility, and compliance through digital technologies. A telerehabilitation program called “Future Patient—telerehabilitation of patients with AF (FP-AF)” has been developed to enhance rehabilitation for AF. The FP-AF program comprises two modules: (1) an education and monitoring module using telerehabilitation technologies (4 months) and (2) a follow-up module, where patients can measure steps and access a data and knowledge-sharing portal, HeartPortal, using their digital devices. Those patients in the FP-AF program measure their heart rhythm, pulse, blood pressure, weight, steps, and sleep. Patients also complete web-based questionnaires regarding their well-being and coping with AF. All recorded data are transmitted to the HeartPortal, accessible to patients, relatives, and health care professionals.

**Objective:**

This paper aims to describe the research design, outcome measures, and data collection techniques in a clinical trial of the FP-AF program for patients with AF.

**Methods:**

This is a multicenter, mixed methods, randomized controlled trial. Patients are recruited from AF clinics serving the North Jutland region of Denmark. The telerehabilitation group will participate in the FP-AF program, while the control group will follow the conventional care regime based on physical visits to the AF clinic. The primary outcome measure is AF-specific health-related quality of life, to be assessed using the Atrial Fibrillation Effect on Quality-of-Life Questionnaire. Secondary outcomes are knowledge of AF; measurement of vital parameters; level of anxiety and depression; degree of motivation; burden of AF; use of the HeartPortal; qualitative exploration of patients’, relatives’, and health care professionals’ experiences of participating in the FP-AF program; cost-effectiveness evaluation of the program; and analysis of multiparametric monitoring data. Outcomes are assessed through data from digital technologies, interviews, and questionnaires.

**Results:**

Patient enrollment began in January 2023 and will be completed by December 2024, with a total of 208 patients enrolled. Qualitative interviews conducted in spring 2024 will be analyzed and published in peer-reviewed journals in 2025. Data from questionnaires and digital technologies will be analyzed upon study completion and presented at international conferences and published in peer-reviewed journals by the fall of 2025.

**Conclusions:**

Results from the FP-AF study will determine whether the FP-AF program can increase quality of life for patients with AF and increase their knowledge of symptoms and living with AF in everyday life compared to conventional AF care. The cost-effectiveness evaluation will determine whether telerehabilitation can be a viable alternative for rehabilitation of patients with AF.

**Trial Registration:**

ClinicalTrials.gov NCT06101485; https://clinicaltrials.gov/study/NCT06101485

**International Registered Report Identifier (IRRID):**

DERR1-10.2196/64259

## Introduction

Atrial fibrillation (AF) is a chronic cardiovascular condition occurring in 3% of the adult population, the prevalence of which is predicted to more than double over the next 20 years [1]. The increase in AF prevalence may be attributed to better opportunistic screening for silent AF, aging of the population, and an increase in conditions predisposing to AF, such as obesity, hypertension, diabetes, obstructive sleep apnea, and physical inactivity [2]. If untreated, AF is associated with a 5-fold increase in the risk of stroke; 20%-30% of all strokes are due to this type of arrhythmia [1,3].

AF imposes a substantial economic burden on the health care system, with 10%-40% of patients with AF being acutely hospitalized each year [1]. AF patients may experience a variety of symptoms, such as palpitations, fatigue, dyspnea, chest pain, sleeping difficulties, fear, and anxiety, although there is great variation in the individual symptom levels. While up to 40% of patients with AF are asymptomatic, others report severe or disabling symptoms [1]. Sleeping difficulties such as sleep apnea are also observed and may be associated with increased cardiovascular risk, further underscoring the importance of AF management [4,5]. Patients with AF trying to live with their disease can benefit from some form of effective, prolonged, and specialized cardiac rehabilitation (CR).

CR is an outpatient chronic disease management intervention that includes structured exercise. CR is based on a risk-factor assessment combined with health management interventions. Patients are educated and encouraged to quit smoking, attend vocational and nutritional counseling sessions, and seek psychosocial support. A review of CR aimed at patients with AF found gaps in the CR research, notably the need for more rigor in the reporting of intervention details, outcomes, and gender-based characteristics of CR and its effectiveness for patients with AF [6]. Many patients with AF do not feel that they have received sufficient knowledge regarding how to live with their disease [7], while other studies have shown that patients with AF often have poor knowledge about arrhythmia, its treatment, and self-management strategies [6]. In 2019, Denmark published the first national guidelines for CR for patients with AF [8], but actual rehabilitation programs have not yet been implemented. A new, innovative alternative to CR is cardiac telerehabilitation (CTR), in which certain elements of the CR program take place in the patient’s home with the help of wearables and other technologies, such as a blood pressure monitor, and using remote communication between the patient and the health care professionals (HPs) [9]. Continuous monitoring, combined with the use of oral anticoagulation for stroke prevention and maintenance of normal sinus rhythm, has demonstrated benefits such as reduced hospitalization, limiting disease progression, and improving survival [10]. Reviews focusing on digital patient education for patients with AF found improvements in patient knowledge of AF [11]. For example, Desteghe et al [4] found that tailored web-based education was an effective strategy for improving AF knowledge and that patients were positive about using web-based patient education. These findings are in line with a review by Fredericks and Yau [12], who found that individualized patient education in heart surgery patients increases quality of life (QoL), improves performance of health behavior, and ameliorates psychological distress [12].

Psychological distress, such as anxiety and depressive symptoms, is prominent among patients with AF, with prevalence rates ranging from 28% to 38% [13]. Some studies have shown that AF may be a causal factor in the onset of depression, while a few studies find that depression may also cause AF [14-18]. Both anxiety and depression have been associated with recurrent AF [19], while anxiety is also described as a side effect of living with AF [15,20]. Overall, psychological distress has been associated with poorer QoL [19,21] and increased severity of AF symptoms [13,19], whereas QoL has been associated with AF severity [19]. This association highlights the importance of examining the impact of both AF severity and psychological distress on QoL. It should be noted that psychological distress may also impair the patient’s motivation for engaging in rehabilitation, thus reducing participation in rehabilitation and self-care [22,23], whereas successful alleviation of AF symptoms is often associated with reduced emotional distress [24].

The educational CTR program “Future Patient—telerehabilitation of patients with AF” (FP-AF) was developed in Denmark through a cocreation process with patients with AF, their relatives, and researchers. The program was evaluated in a pilot study [25], which showed that patients with AF and their relatives found the FP-AF useful because it created an increased sense of security, increased knowledge about mastering symptoms, and a community of practice linking patients with AF and their relatives with HPs. These findings suggest that there is a potential for increasing the QoL of patients with AF by providing educational telerehabilitation programs that can permit sufficient individualization for patients (and relatives). Following the pilot study, the intention is for the FP-AF program to be evaluated in a multicenter, mixed-methods, randomized controlled trial (RCT). The development of the program is in line with the strategies of user involvement developed by the Danish Heart Association and with national strategies promoting digital health in Denmark, as well as current recommendations for AF rehabilitation [8].

The purpose of the FP-AF program (ClinicalTrials.gov NCT06101485) is to increase the QoL of patients with AF by providing both patients and their relatives additional knowledge about AF and practical guidance on how to live with AF in everyday life. The program aims to individualize the rehabilitation process by helping patients and their relatives develop their own self-management strategies using their own clinical data and enhanced knowledge about AF. This paper describes the research design, outcome measures, and data collection techniques used in the FP-AF research project.

## Methods

### Research Design

The FP-AF program will be evaluated in a multicenter, mixed methods, RCT study. The telerehabilitation group will participate in the FP-AF program, while the control group will follow the conventional care regime based on physical visits to the AF clinic. Enrollment of patients began in January 2023, and the RCT is expected to end in June 2025.

### Ethical Considerations

The Future Patient project has been approved by The Scientific Ethics Committee for the North Denmark Region (N-20220056). The study is listed in ClinicalTrials.gov (NCT06101485). The study is being carried out in accordance with the Helsinki Declaration, and all participants have signed an informed consent form prior to enrollment in the study. Participants have been informed that they have the right to withdraw their consent at any point in time during the study, and the reason for their withdrawal will also be documented if the participant so wishes. The participants have not received any compensation for their participation in the study. Upon the randomization, all patients have been assigned an individual identification number so their data remain anonymous. The project team will then collect the equipment upon request.

### Procedure and Eligibility Criteria

All patients diagnosed with AF at the Departments of Cardiology at Central Region Hospitals in Viborg, Skive, and Silkeborg will be assessed for eligibility in the study. The inclusion criteria are as follows: patients must be diagnosed with AF; they must be adults aged 18 years or older; they must live in Skive, Viborg, or Silkeborg municipalities; they must live at home and be capable of caring for themselves; and they must possess basic computer skills or have a relative or friend with basic computer skills.

Exclusion criteria are as follows: pregnancy; refusal or inability to cooperate; patient does not speak, read, or understand Danish; and patient has a life expectancy of less than a year.

A CONSORT (Consolidated Standards of Reporting Trials) diagram will be used to document all data pertaining to the recruitment process, as well as patient withdrawal or dropout.

### Power Calculation

The primary outcome of this study is AF-specific health-related QoL (HRQoL) as measured by the Atrial Fibrillation Effect on Quality-of-Life Questionnaire (AFEQT) through the use of telerehabilitation compared to conventional rehabilitation. A clinically significant mean difference of 11 points between the intervention and control groups and an SD of 23 points were used in the study power calculation, based on prior literature [26]. A sample size of 184 patients would have 90% power to demonstrate a difference between the groups. This project estimates a dropout rate of about 10%. Thus, the total number of patients required in the study is 208, with 104 patients in the intervention group and 104 in the control group.

### Randomization

Randomization of the patients will be conducted according to a randomized block design using random sized blocks of 4, 6, and 8 patients. The blocks will be stratified by center and appropriate age group and sex. The randomization will be digital and blinded for the project nurses who will be recruiting patients for the study.

### Theoretical Framework

The FP-AF program is based upon self-determination theory (SDT), which describes motivation as an essential part of any successful rehabilitation [27]. SDT describes human motivation as the fulfillment of 3 basic needs: autonomy, competency, and relatedness. To ensure continuous motivation, it is necessary that this motivation is intrinsic to the patient, which requires that the patient feels that all 3 of these primary needs are being supported simultaneously [27]. As such, intrinsic motivation is achieved when a patient (1) experiences autonomy, that is, identifies with the goals of rehabilitation and values these as personally important; (2) experiences competency, that is, the patient believes that he or she has the necessary knowledge and skills to achieve the goal and receives guidance and feedback; and (3) experiences relatedness, that is, the HP’s and the patient’s social network create a milieu in which the patient feels respected, understood, and supported.

### Future Patient Telerehabilitation Program

The FP-AF education program for patients with AF and relatives consists of two modules: (1) an education and monitoring module using telerehabilitation technologies (4 months) and (2) a follow-up module, where the patients use their own personal devices to measure steps and to access the HeartPortal (3 months).

After enrollment, those patients randomized to the intervention group meet individually with the project nurse. At this meeting, the patient (and relatives, if necessary) are instructed in the use of the technologies (see details below), and an individual plan is formulated for the AF patient’s telerehabilitation program. The project nurses at the AF clinics (Regional Hospitals in Viborg and Silkeborg) review all the patients’ measured values twice a week and have continuous contact with the patients during their enrollment in the project. For each patient, the physicians at the hospital set limit values for blood pressure, pulse, and weight. If patients register values that are out of range, they are instructed to contact the project nurse or the cardiology ward. Furthermore, the patients and relatives are invited to participate in an educational module at the local health care center, where offerings include topics such as living with AF, symptoms of AF, management of own disease using digital technologies, and how you as a relative can support your spouse or partner.

### The HeartPortal

**Figure 1:**
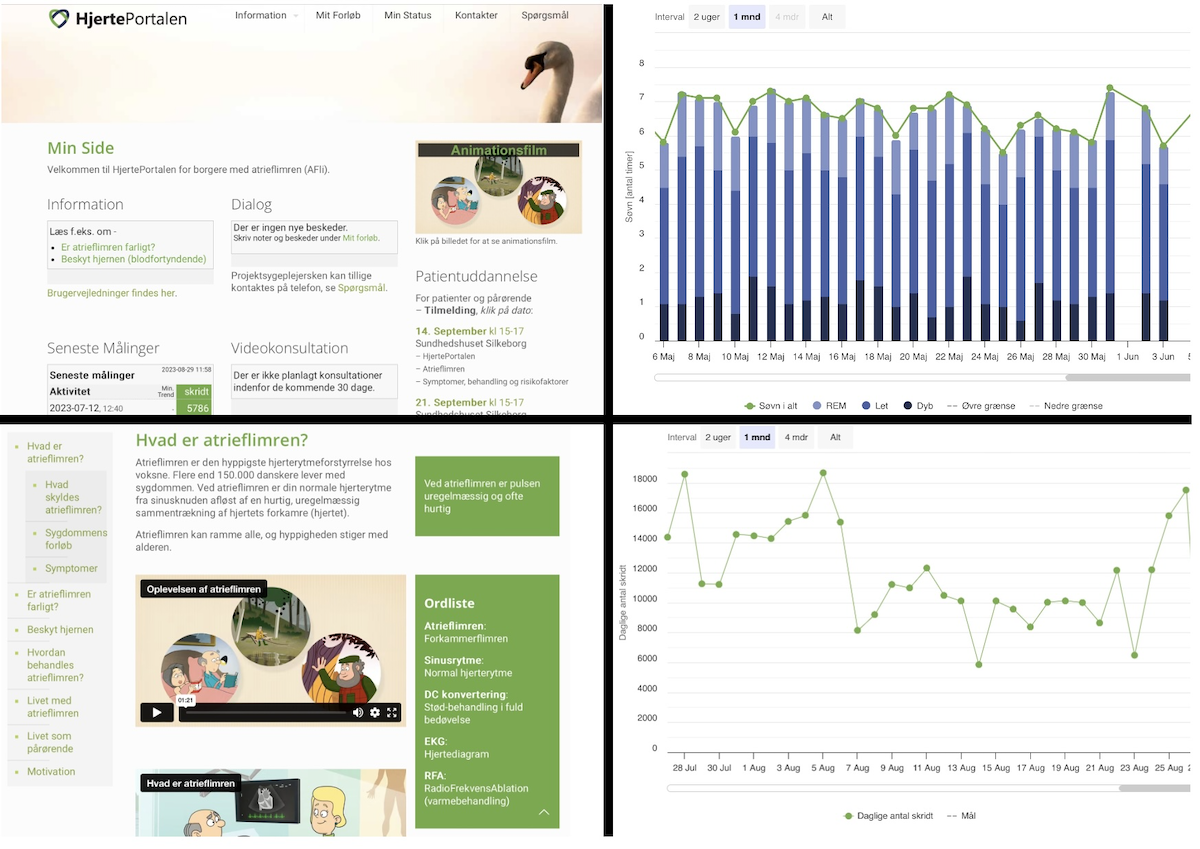
Screen captures from the HeartPortal. (A) The patients front page. (B) Measurement from the sleep sensor. (C) The information platform. (D) Measurement from the pedometer.

**Figure 2:**
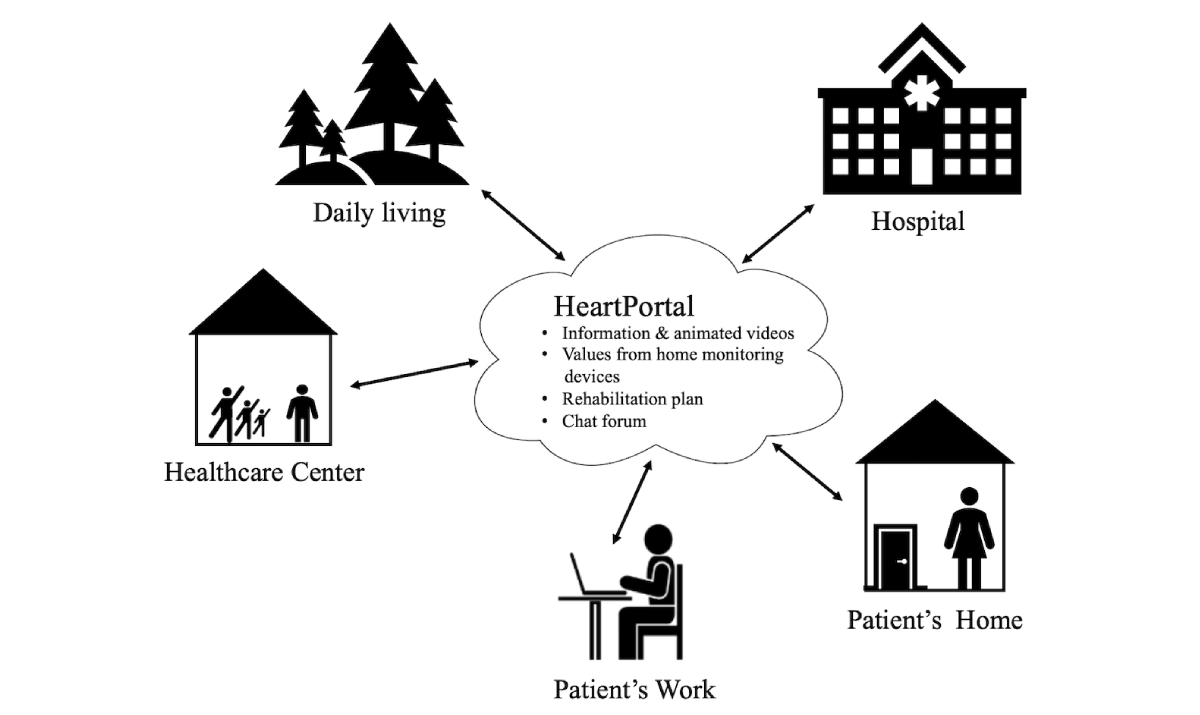
The context of the Future Patient program.

The HeartPortal (version 1.0), a web-based portal used by patients, relatives, and HPs at the hospitals and health care centers, functions as a digital toolbox and learning module. Patients and relatives in the telerehabilitation group will have access to the HeartPortal, where they can read texts and watch animations about AF, have access to visualization of the patients’ measured data (steps, weight, pulse, blood pressure, and sleep), and communicate in video and chat with HPs at the hospitals and health care centers. The patients can access the data from the electrocardiogram (ECG) on a separate platform. Screen captures of the HeartPortal are shown in Figure 1, and the context of the Future Patient study is shown in Figure 2.

The HeartPortal consists of 4 elements (Textbox 1).

The technologies being used in the CTR group are listed in Textbox 2.

**Four elements of HeartPortal.**

- A platform containing atrial fibrillation and rehabilitation related information pages (text, images or illustrations, animation, link to other validated sources, etc).
- Visualization of measured values (steps, weight, pulse, blood pressure, and sleep) in graphs that offer an easy overview and trends in data.
- A communication platform (video and chat) that enables patients to communicate directly with health care professionals. Video consultations will be conducted at 1 month and 4 months after enrollment in the study and additionally as needed on an individual basis.
- A rehabilitation plan. For each patient, an individualized rehabilitation plan is defined in a dialogue between the patient, the doctor, and a project nurse. The rehabilitation plan is formulated using individual parameters such as weight, physical status, pulse, and blood pressure. The patients write down their personal rehabilitation goals on the HeartPortal. The patients start the rehabilitation plan at the time of enrollment in the study. A project nurse monitors the patient’s data twice a week and contacts the patients if the rehabilitation goals have not been met, data have not been transmitted to the HeartPortal, or in case data are not within the normal range set for the patient.

**Technologies used in the cardiac telerehabilitation group.**

- Blood pressure monitor (iHealth Neo [BP5S]) [28]. Measurement of systolic and diastolic pressure and pulse. Connects to an app on the tablet to record the measurement and the tablet’s internet connection to transfer the measured values. Blood pressure is measured twice a week.
- Weight scale (iHealth Lina) [29]. Allows the patient to follow his or her weight and observe weight loss or gain. Connects to an app on the tablet to record the weight, and the tablet’s internet connection transfers the measured values to the HeartPortal. Weight is measured twice a week.
- Activity tracker (Fitbit Inspire 3|Health & Fitness Tracker) [30]. Measures physical activity, counted as steps and calories burned as well as pulse throughout the day. The activity tracker can also measure sleep if worn while sleeping. An app on the tablet is used for viewing and reviewing the tracked data and for data transfer. Activity, measured as steps, is measured every day and transmitted to the HeartPortal.
- Electrocardiography (ECG) monitor (KardiaMobile) [31]. Consists of a small device with touchpads, one for each hand. The monitor uses ultrasound to communicate with an app on the tablet. The app transfers the data to a General Data Protection Regulation-compliant system for clinicians, KardiaPro. ECG is carried out twice a week. The Kardia device includes an artificial intelligence–powered algorithm with an accuracy rate of 97% [31]. When patients measure their ECG, the device detects their heart rhythm. Patients have also been taught to interpret the results, such as the difference between sinus rhythm and AF.
- Sleep sensor (Emfit QS) [32]. Monitors pulse and respiration during sleep, sleep duration, stages, and sleep quality. Sleep is measured in hours every night, and the sensor data are transmitted to the HeartPortal.
- Video solution (VDX, Medcom) [33], a national video platform in Denmark within health care.

### Data and Network Security

The telerehabilitation program has a strong focus on data and network security and resiliency. Security and resiliency in eHealth is a general issue that requires special measures in an environment where data are generated both from a health system (pulled data) and from the users or patients (pushed data). The format of metadata for reliable integration (source traceability), encryption, and infrastructure reliability (VPNs, VLAN, centralized vs federated storage) will be addressed by including perspectives from the different endpoints in the system (home, workplace, health center, etc), the technology used (wired, wireless, and dual technology), and the classification of information. Standardization activities, such as those from ITU-T, Continua, ENISA, etc, will be monitored and applied where possible.

### Usual Care Intervention

Patients in the control care group have received AF education as usual, with the standard 45-60 minutes of orientation in AF, symptoms, treatment, and management of their own disease delivered in person at the hospital and by consultancy if needed by their general practitioner.

### Baseline Characteristics

Sociodemographic data, such as civil status, level of education, work status, and IT competences, will be collected from both groups at baseline using data from the patient’s electronic medical record and questionnaires.

Clinical baseline data, such as clinical status, primary and secondary diagnoses, and prescribed medicine, will also be collected for both groups using the electronic patient record.

### Outcome Measures

Table 1 presents an overview of the primary and secondary outcome measures, along with the scheduled data collection dates. Except for progression in clinical data, including adverse events and dropout, all outcome measures will be collected using packages of questionnaires relevant to the specific point in time. The patients answer questionnaires in a web-based version, or on paper if they prefer.

**Table 1:** Primary and secondary outcome measures and data collection dates.

Outcomes		Time of measurement					End of tele- rehabilitation (at 4 months)		Follow-up at 7 months
		Baseline	Every day	Twice a week	As needed				
**Primary**									
	Changes in AF^a^-specific quality of life	(I^b^,C^c^)				(I,C)		(I,C)	
**Secondary**									
	Changes in AF knowledge	(I,C)				(I,C)		(I,C)	
	*Measurement of vital parameters*								
	Weight	(I,C)		(I)	(I)				
	Blood pressure	(I,C)		(I)	(I)				
	Pulse	(I,C)	(I)		(I)				
	Steps	(I)	(I)			(I)		(I)	
	Sleep	(I)	(I)						
	Electrocardiogram	(I,C)		(I)	(I)				
	Changes in anxiety and depression	(I,C)				(I,C)		(I,C)	
	Changes in motivation	(I,C)				(I,C)		(I,C)	
	Burden of AF	(I,C)				(I,C)		(I,C)	
	Use of HeartPortal					(I)			
	Patients’ and relatives’ perspective and experiences					(I)			
	Health care professionals’ perspective and experiences					(I)			
	Cost-effectiveness evaluation					(I,C)		(I,C)	
	Trends and patterns in multi-parametric clinical data					(I)			

^a^AF: atrial fibrillation.

^b^I***:*** intervention group.

^c^C: control group.

#### Primary Outcome

##### Changes in AF-Specific Health-Related Quality of Life

AF-specific HRQoL is measured using the AFEQT [34]. The AFEQT is a 20-item self-administered questionnaire designed to assess the impact of AF on the patients’ HRQoL across symptoms, daily activities, treatment concerns, and treatment satisfaction. Scores range from 0 to 100, with higher scores indicating better HRQoL.

#### Secondary Outcomes

##### Changes in AF Knowledge

The Jessa Atrial Fibrillation Knowledge Questionnaire (JAKQ) is a 16-item scale used to assess the knowledge of patients’ AF about the arrhythmia illness [4]. For every item, false responses or “I don’t know” responses are scored as 0, and the correct responses are scored as 1. The total score of the scale is calculated by adding up all item scores.

##### Measurement of Vital Parameters

Clinical data on weight, blood pressure, pulse, steps, sleep, and ECG in the intervention group will be collected as specified in Table 1.

##### Changes in Anxiety and Depression

Symptoms of anxiety and depression are measured using the Hospital Anxiety and Depression Scale (HADS) [35]. The HADS is a 14-item self-reported questionnaire often used for screening of psychological distress in patients with cardiac conditions. Each item is scored on a 4-point Likert scale ranging from 0 to 3. The 2 subscales, anxiety and depression, each comprise 7 items, and scores on these subscales range from 0 to 21, with higher scores indicating higher levels of anxiety or depressive symptoms.

##### Changes in Motivation

Motivation is measured by the Health-Care Self-Determination Theory Questionnaire Packet (HC-SDTQ). The HC-SDTQ includes 3 separate questionnaires: the Treatment Self-Regulation Questionnaire, which focuses on autonomy as stipulated by SDT [36]; the Perceived Competence Scale, which assesses patients’ sense of competence about engaging in, or maintaining, a healthier behavior [37]; and the Health Care Climate Questionnaire, which assesses the patient’s perception of autonomy support from their health care provider [38].

##### Burden of AF

The burden of AF is measured using the Arrhythmia-Specific Questionnaire in Tachycardia and Arrhythmia (ASTA) [39]. The ASTA measures symptoms and HRQoL in patients with different forms of arrhythmias, including AF. The ASTA is divided into 3 parts: Part I assesses demographic data, such as the most recent episode of arrhythmia, current medication, and the presence of arrhythmia at the time of follow-up; Part II measures the arrhythmia-specific symptom burden; and Part III measures HRQoL. Parts II and III are scored using a 4-point Likert-type scale (0=“No” to 3=“Yes, a lot”). The total score range is from 0 to 100, with a higher score indicating a higher symptom burden or a negative effect on HRQoL.

##### Use of HeartPortal

Time log files for login and logout of patients and relatives will be analyzed in order to identify which parts of the HeartPortal are being used and for how long. The patients will be asked for their consent prior to extracting their log files from the database for analysis.

##### Perspectives and Experiences From Participating in the FP-AF Program

Participant-observation [40,41] will be used in situations such as educating and counseling the patients and relatives in using the technologies at home and to observe how patients and relatives use the digital technologies and the HeartPortal.

Perspectives and experiences of patients, relatives, and HPs participating in the FP-AF program will be explored using semistructured qualitative interviews, inspired by Brinkmann and Kvale [42]. Interviews will be performed until data saturation is reached, with a minimum of 10 interviews conducted for each group: patients, relatives, and HPs. The interviews will be recorded, transcribed, and analyzed in themes inspired by Brinkmann and Kvale [42] and based on their phenomenological approach. The interviews will be coded by 2 researchers using NVivo (version 14.0) software [43].

##### Cost-Effectiveness Evaluation

Throughout the duration of the study, we will also collect information on unplanned admissions for any reason with at least 1 overnight stay, unplanned readmissions for any reason and length of stay, visits to outpatient clinics, rehabilitation activities, visits to the general practitioner, equipment used, driving distance, personnel use, and numbers of phone or video calls. The analysis will be based on the guidelines for economic evaluation elaborated by Drummond et al [44].

### Data Storage

Demographic data are being stored within the Future Patient database, and the questionnaire data are stored in the REDCap (Research Electronic Data Capture; Vanderbilt University) software for quantitative analysis.

### Adverse Events, Dropout, and Technical Issues

All adverse events, dropouts, and deaths, as well as any technical issues related to the equipment, will be registered. No preterm study stop criteria are planned.

### Data Analysis

#### Statistical Analysis

Baseline characteristics will be presented as frequencies and percentages or as medians and IQRs, as appropriate, and the data will be stratified by randomization groups and by sex. Changes over time on the primary outcome will be analyzed in accordance with the intention-to-treat principle, using a mixed-effects model for repeated measures design with robust variance in order to relax the assumption of normally distributed residuals. In addition, margin plots will be produced in order to investigate the interaction of time and randomization group. A sensitivity analysis will be conducted in order to investigate the effects of missing data as a result of dropout by comparing baseline characteristics between complete cases and dropouts, followed by adjusted analyses of the primary outcome using appropriate baseline characteristics as adjustment parameters. Secondary outcomes will be analyzed in a manner similar to the primary outcome. We will also examine overlaps between and variations within groups of different sexes. In examining sex differences, we will adjust for possible confounding factors (eg, age).

#### Trends and Patterns in Multiparametric Clinical Data

Data will be analyzed in order to identify correlations in multiparametric data collected during the 4 months. Initial analysis of the multiparametric data will be carried out by assessing the architectural structures and complexities of the data design through basic statistical and distributive analysis. Differences in the multiparametric data between baseline and postintervention measurements will be tested with paired *t* tests. Correlations of multiparametric data will be compared with patient-reported symptoms of AF. Paired *t* tests will be performed for the multiparametric data between periods both with and without patient-reported symptoms of AF in patients experiencing symptoms. Also, *t* tests will be performed for multiparametric data between patients with and without symptoms of AF. Simplistic features will be developed based on the statistical analyses and correlations between data.

#### Qualitative Analysis

A research assistant will transcribe all interviews into text files. Data will be coded in NVivo (version 14.0; QSR International), drawing inspiration from the SDT theoretical framework and using methods developed by Brinkmann and Kvale [42]. Two different researchers will be responsible for conducting the interviews and analyzing the data. The data will be presented in terms of key themes and major findings.

#### Economic Evaluations

A cost-effectiveness analysis of the FP-AF telerehabilitation program compared to conventional care will be made at 7 months after inclusion for both groups. The analysis will be based on the guidelines for economic evaluation elaborated by Drummond et al [44].

## Results

The findings of the RCT will be analyzed during the spring of 2025 and subsequently published in peer-reviewed journals in the fields of telerehabilitation, clinical cardiology, and health economics. In addition, these results will be presented at relevant international scientific conferences. A total of 208 patients will be enrolled, 104 in the telerehabilitation group and 104 in the control group.

The evaluation of the FP-AF program comprises both clinical data recorded by the patients and data gathered through interviews and questionnaires. The evaluation involves quantitative analysis of primary and secondary outcomes (questionnaires) as well as qualitative exploration of the perspectives and experiences of patients with AF, their relatives, and HPs. Finally, a health economic evaluation will be conducted. Taken together, the data and the associated analyses will provide a comprehensive perspective on the effectiveness of the FP-AF program as seen from clinical, patient, relatives, psychological, organizational, and economic perspectives.

## Discussion

### Telerehabilitation Program

The aim of the study is to test an individualized CTR program for patients with AF. The primary outcome measure is to assess potential changes in QoL among those patients with AF using telerehabilitation. The secondary outcome measures are changes in AF knowledge, changes in anxiety and depression, changes in motivation, the burden of AF, patients’ and relatives’ perspectives and experiences, patients’ and relatives’ use of the HeartPortal, perspectives and experiences of HPs, the development of clinical data, and a cost-effectiveness analysis.

CR for patients with AF is a fairly new initiative, and national guidelines for AF CR have only recently been published [8]. In this study, we take this initiative further by examining the potential of a CTR program for patients with AF, as telerehabilitation provides a promising and innovative alternative to conventional rehabilitation. The potential advantage of telerehabilitation is that it can provide easier access to rehabilitation assistance and activities and may therefore be a means of overcoming some of the barriers to participation in CR that have been identified in other cardiac patient groups [45]. Use of telerehabilitation may be especially relevant for patients with AF, as this group is very diverse in terms of the subjective impact of their disease; hence, some patients may not feel the need to attend physical appointments at the clinic, but they may be willing or even positive about participating through digital communication and technology. The digital option offered by telerehabilitation may thus improve their QoL.

The FP-AF program will be tested in a multicenter study in order to increase the validity of the study. The FP-AF program is based upon SDT theory and the exploration of how patients, their relatives, and HPs use the HeartPortal. We will therefore analyze the linkages between motivation and use of the digital platform. In a previous study of patients with heart failure, we have documented that telerehabilitation motivates patients with heart failure just as effectively as conventional rehabilitation and that it does not lead to higher levels of psychological distress [46].

To obtain a more in-depth understanding of how patients, relatives, and HPs perceive CTR, we also plan to conduct qualitative interviews with all groups in order to learn more about how they experience the program as part of their everyday life and work, including their evaluation of the specific modules of the program. Using interviews together with questionnaire data will allow for a more holistic understanding of the usability, effect, and acceptance of the proposed program.

### Limitations

The expected limitations for the interpretations of the results are as follows. The study relies heavily on commercial tracking devices that have been chosen for their ability to automatically transmit data to the HeartPortal. Any changes in device regulations during the study could make it necessary to replace these devices, potentially leading to inconsistency in the data. In addition, the proposed intervention is complex, consisting of a chain of events and interventions and based on multiple commercially available technologies that may become outdated. This complexity, including the rapidly changing nature of technology, may pose a challenge to future replication studies.

### Conclusions

Analyzing data from primary and secondary outcomes will help determine whether the FP-AF program has the potential to increase QoL for patients living with AF and give them more knowledge of how to master the disease. The cost-effectiveness analysis will provide an assessment of the health economic sustainability of the FP-AF program and shed light on whether telerehabilitation can be a viable alternative for rehabilitation of patients with AF.

## Abbreviations

AF: atrial fibrillation
AFEQT: Atrial Fibrillation Effect on Quality-of-life Questionnaire
ASTA: Arrhythmia-Specific Questionnaire in Tachycardia and Arrhythmia
CONSORT: Consolidated Standards of Reporting Trials
CR: cardiac rehabilitation
CTR: cardiac telerehabilitation
ECG: electrocardiogram
FP-AF: Future Patient—Telerehabilitation of patients with atrial fibrillation
HADS: Hospital Anxiety and Depression Scale
HC-SDTQ: Health-Care Self-Determination Theory Questionnaire
HP: health care professional
HRQoL: health-related quality of life
JAKQ: Jessa Atrial Fibrillation Knowledge Questionnaire
QoL: quality of life
RCT: randomized controlled trial
REDCap: Research Electronic Data Capture
SDT: self-determination theory
